# Combating trade in illegal wood and forest products with machine learning

**DOI:** 10.1371/journal.pone.0311982

**Published:** 2025-01-24

**Authors:** Debanjan Datta, John C. Simeone, Amelia Meadows, Willow Outhwaite, Hin Keong Chen, Nathan Self, Linda Walker, Naren Ramakrishnan

**Affiliations:** 1 Department of Computer Science, Virginia Tech, Arlington, VA, United States of America; 2 Simeone Consulting, LLC, Littleton, NH, United States of America; 3 World Wildlife Fund, Washington, DC, United States of America; 4 TRAFFIC, Cambridge, United Kingdom; University of Bucharest, ROMANIA

## Abstract

Trade in wood and forest products spans the global supply chain. Illegal logging and associated trade in forest products present a persistent threat to vulnerable ecosystems and communities. Illegal timber trade has been linked to violations of tax and conservation laws, as well as broader transnational crimes. The United States is the largest importer globally of wood and forest products, such as pulp, paper, flooring, and furniture—importing $78 billion in 2021. Transaction-level data such as shipping container manifests and bills of lading provide a comprehensive data source that can be used to detect and disrupt trade that may be suspected of containing illegally harvested or traded forest products. Owing to the volume, velocity, and complexity of shipment data, an automated decision support system is required for the purposes of detecting suspicious forest product shipments. We present a proof of concept framework using machine learning and big data approaches—combining domain expertise with automation—to achieve this objective. We formulated the underlying machine learning problem as an anomaly detection problem and collected and collated forest sector-specific domain knowledge to filter and target shipments of interest. In this work, we provide the overview of our framework, with the details of domain knowledge extraction and machine learning models, and discuss initial results and analysis of flagged anomalous and potentially suspicious records to demonstrate the efficacy of this approach. The proof of concept work presented here provides the groundwork for an actionable and feasible approach to assisting enforcement agencies with the detection of suspicious shipments that may contain illegally harvested or traded wood.

## 1 Introduction

Wood and forest products, like furniture, are valuable, globally traded commodities. Like the international trade of many other highly valued natural resources, the trade in forest products faces challenges, including corruption, fraud, and the laundering of illegally harvested wood [1–3]. These acts are not limited to the country where the wood was harvested but extend throughout the global supply chain and have been tied to illicit financial flows (e.g., trade-based money laundering), document fraud, species mislabeling, and other suspicious, high-risk, and potentially illegal activities [4–8]. Illegal logging is the third largest transnational crime, after counterfeiting and drug trafficking, and is the most profitable natural resource crime, with an estimated annual value between $52 billion and $157 billion [9]. However, at each node of the global supply chain of forest products, identifying suspicious shipments potentially linked to illegal activities is a persistent challenge faced by law enforcement agencies.

**Fig 1:**
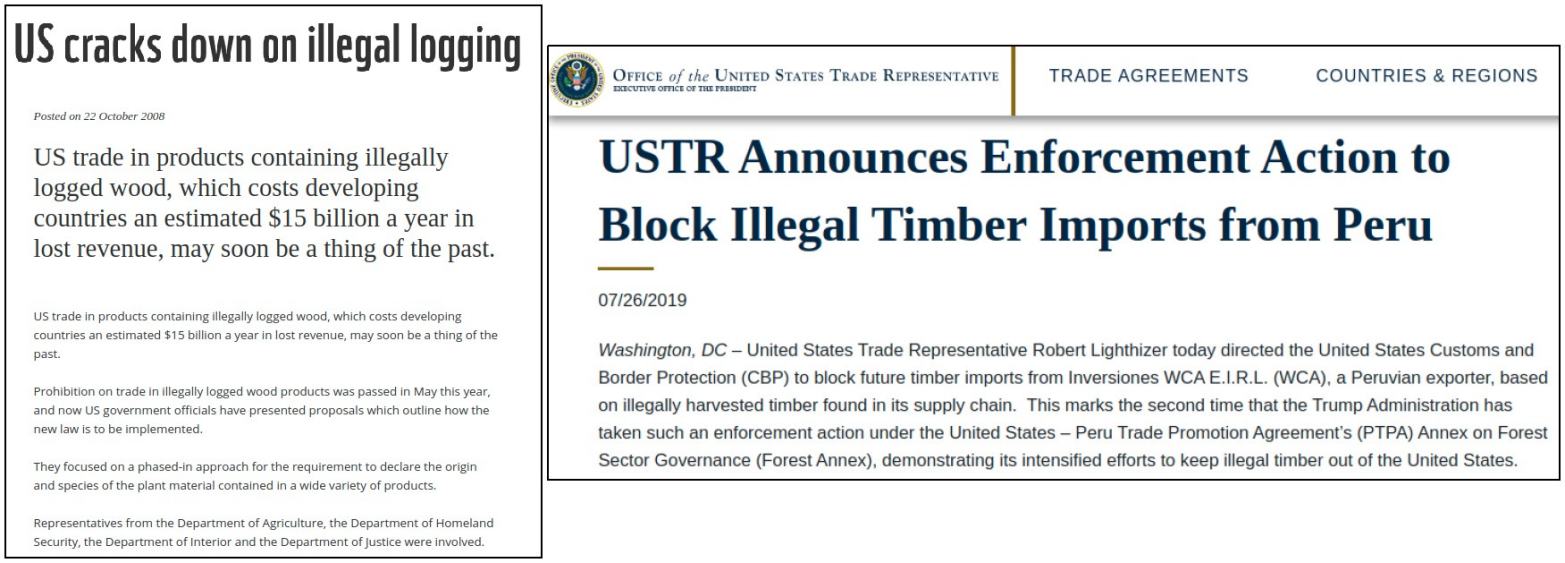
Example press releases noting the US government’s prioritization of combating illegal wood imports: (L) World Wildlife Fund (WWF), (R) office of the US Trade Representative (USTR).

The United States (US) is the largest importer of wood and forest products globally, with imports growing each year. In 2017, US imports totaled $51 billion—representingover 20% of the global trade in forest products, and in 2021 US imports reached $78 billion [8]. In 2008, the US amended the US Lacey Act and extended its provisions to make it unlawful to import any plant or plant product that was illegally harvested (18 U.S.C.§42–43; 16 U.S.C.§3371–3378). The 2008 Lacey Act Amendment also set forth a Plant and Plant Product Declaration requirement which requires importers of specified products, as identified by their 6-digit Harmonized Schedule (HS) codes and their 10-digit US Harmonized Tariff Schedule (HTS) codes, to declare the scientific name and country of harvest of all wood species contained in the product [10].The HS and HTS code nomenclature are described in more detail in Section 3 (Methodology). In addition to the Lacey Act, the US is party to CITES, the Convention on International Trade in Endangered Species of Wild Fauna and Flora (27 U.S.T. § 1087). By implementing and enforcing these two statutes, the US government has the authority to combat the role it plays in contributing to illegal logging and associated trade. The US government has taken this task seriously and has prosecuted several high profile Lacey Act cases as well as taken enforcement actions to block imports of forest products suspected of being illegally harvested and traded—see Fig 1 [11–17].

Nevertheless, detecting, deterring, and enforcing trade in illegally harvested wood can be difficult for US government agencies due to (*i*) volume, velocity, and complexity of trade records; (*ii*) short investigative window and high cost to detain cargo; and (*iii*) a paucity of forest sector specific tools for targeting and long term trend analysis. Illegally harvested wood and associated trade can be difficult to identify, particularly when it is packed into shipping containers. At present, it is critical for border law enforcement staff to be familiar with red-flags associated with suspicious shipments so they know when to inspect and interdict, such as when shipping routes appear to be incongruous with goods declared, vague information on documents, discrepancies between information declared and the weight, value, or appearance of the shipment. While manual examination and partially algorithmic tools are utilized by agencies, an integrated framework to highlight suspicious shipments is not readily available. This can be attributed to factors which include: (*i*) hurdles in policy implementation [18]; (*ii*) inter-agency communication and information-sharing agreements; (*iii*) handling how non-governmental organizations and government agencies share sensitive information that might lead to an investigation and prosecution [19]; (*iv*) lack of unified domain knowledge that can be incorporated into such systems; and (*v*) the absence of expert-annotated data to adopt off-the-shelf machine learning models.

Identifying specific US imports that may be at higher risk of containing illegally harvested wood can allow US government agencies to target which shipments need additional scrutiny, and to determine patterns of suspicious trade involving specific companies and trade routes. To achieve a near real-time process that is actionable by enforcement agencies, however, requires an automated framework to detect potentially suspicious shipments of forest products given the volume and velocity of trade data. Developing algorithms that identify potentially suspicious trade is a non-trivial task, exacerbated by the size of shipment data as well as the lack of ground-truthed positive training data—that is, data on shipments that are found to have confirmed illegalities, as well as the challenges associated with obtaining what data does exist from government and enforcement agencies given information sharing constraints and potential impacts to ongoing investigations.

Shipment-level US import *Bills of Lading* (BoL) are submitted by importers to the US government and are one of several documents that importers of goods into the US must file with various US government agencies. Data can be purchased from third-party companies, like S&P Global’s Panjiva [20], which provide both the raw data as well as a selection of derived data that can aid interpretation and analysis. Shipment-level BoL data have been shown to be underutilized in research on international trade [21].

Non-governmental organizations use shipment-level BoL data for commodity-specific supply chain risk analyses and it has been a pivotal part of exposing suspicious and illegal tree harvesting and associated forest product trade [22]. One public example is the Environmental Investigation Agency’s use of shipment-level BoL data from US, China, and Russia during their investigation of imports by the US company Lumber Liquidators of illegally-harvested Russian-origin oak that had been manufactured into flooring in China [4]. These uses of trade data led us to consider whether machine learning approaches could assist in the identification of suspicious shipments of natural resources at key supply chain nodes.

## 2 Problem formulation

**Fig 2:**
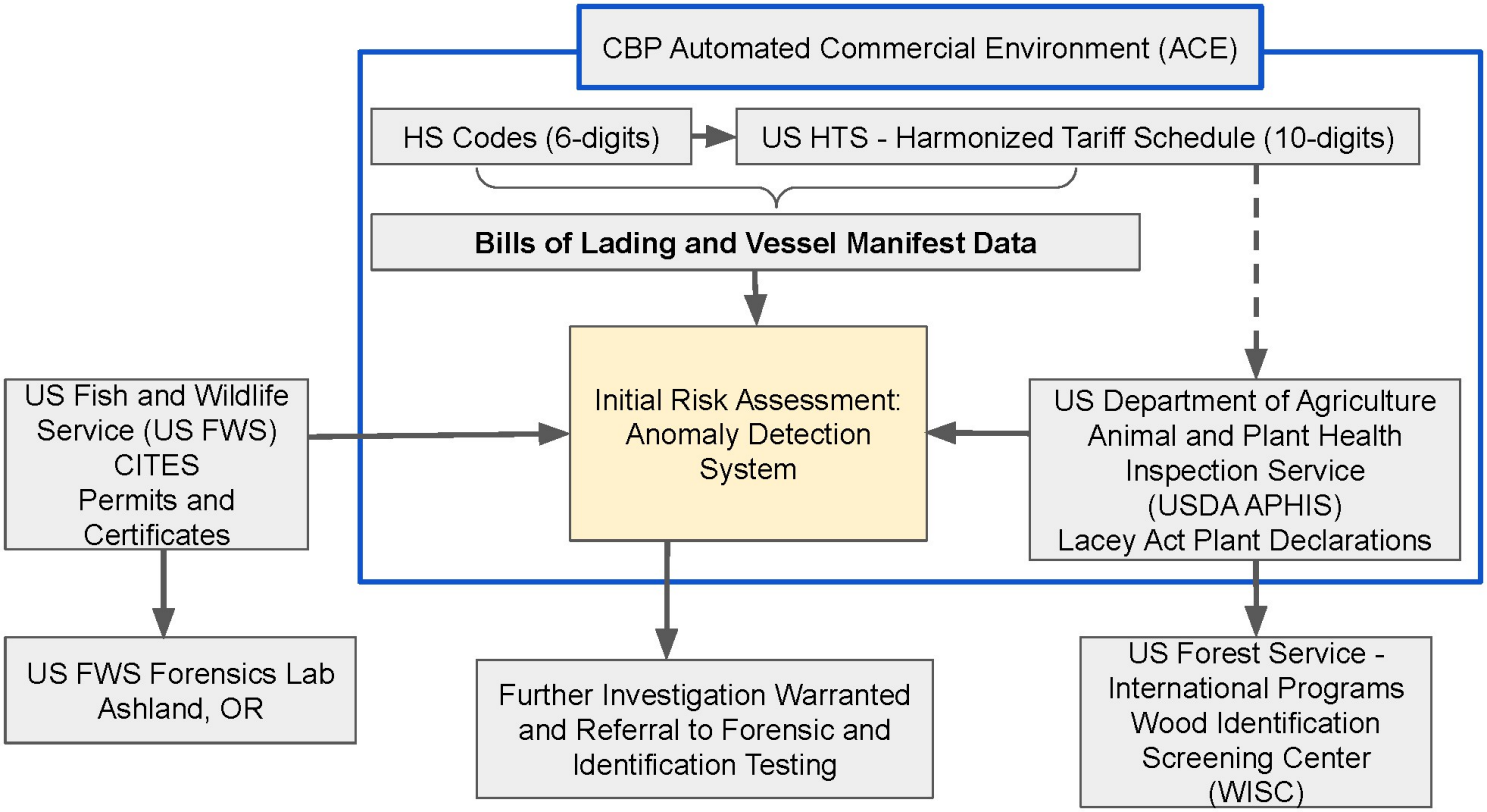
Initial risk assessment: Anomaly detection system context for deployment within US wood and forest product import reporting requirements.

We hypothesized that machine learning could help US agencies flag suspicious imports of forest products with better speed and accuracy, reducing illegal logging and associated impacts on wildlife and people around the globe. We designed our system for US Customs and Border Protection (CBP) as the primary end-user, and thus we made sure that both the inputs and outputs of our system were aligned with the broader regulatory reporting framework for US wood and forest product imports. Fig 2 provides an overview of the regulatory context within which we envisioned our system being deployed.

We set out to develop a proof-of-concept framework and initial risk assessment system for automated discovery of potentially suspicious BoL records, thus aiding US government agencies tasked with Lacey Act and CITES enforcement. Based on a literature review of each of this research’s sub-disciplines—including, using data science approaches to detect suspicious and fraudulent activity [23, 24], natural resource supply chain analysis [25, 26] and trade-based money laundering [27, 28]—a set of assumptions were made in order to formulate our research into manageable, actionable, workflows. One such assumption was that suspicious shipments and shipments consistent with trade in illegally harvested wood do not conform to expected trade patterns. A second assumption we made is that these unexpected trade patterns are not easily recognizable by a human analyst exploring the data, but may be detected by a system that scrutinizes hundreds of thousands, or millions of shipment records. From a data science perspective, we, therefore, formulated the task of identifying potentially suspicious forest product shipments as an anomaly detection problem.

There are multiple challenges in building such a framework, with a key aspect being determining how to connect a human-knowledge component to the overall framework. While human knowledge from experts can not be directly incorporated into an automated decision support system, data sources that encompass this knowledge provide a tractable solution. We formulate this aspect of incorporating human knowledge as *human-defined filters*, which enable matches on data attributes that can be related to high-value wood species that have been previously identified as being high-risk for being illegally harvested and traded using domain knowledge from subject matter experts.

## 3 Methodology

The underlying datasets we utilized were shipment-level BoL trade data covering a three year period from 2015 to 2017, which was purchased from S&P Global Panjiva [20]. We purchased four separate datasets from Panjiva covering identical years—Peru export data, China import data, China export data, and US import data—with each country’s dataset having slightly different attributes in the original BoL data provided to Panjiva.

For instance, China import BoL contains attributes such as *Transport Method and Administrative Region* and Peru BoL contains attributes such as *Transport Method, Vessel Registration, Location Code, and Customs Agent Code*.

However, all the datasets contain key attributes such as the HS Code, albeit with different granularity, as well as trading entities and ports that help define trade routes and patterns. For certain datasets that lack the original official HS code used by the company, like US import data, some BoL data providers, like Panjiva, utilize keyword extraction algorithms that are run on the commodity description field, and reconstitute an assumed 6-digit HS code for each record. Panjiva does try to compute an estimated value of the shipment and provides its own created variable in the US BoL data it sells by calculating average unit values from the original weight/volume given [20, 21].

Despite using historical time series datasets for this proof-of-concept research, we devised a framework that allowed for the potential in the future to run on both historical and real-time BoL data. Almost every country’s commerce that is traded globally by sea requires a BoL to be issued [29], thus indicating that even though we used BoL datasets covering three countries (US, Peru, and China), our framework has the potential to be applied to any country’s BoL trade data. Our framework is made up of modules that encapsulate logic and responsibilities, and work in concert with each other to analyze BoL data, with each module being able to be modified and updated. The framework architecture integrates domain expertise-derived information into the domain knowledge module, that operates with the machine learning module. These two modules provide complimentary functionality in our framework.

### 3.1 Domain knowledge module

We performed an initial formative study involving our collaborating domain experts to understand how to design of our system and how human knowledge can provide key information points. These domain experts with prior experience in dealing with illegal timber trade and forestry, with enforcement agencies. They were well informed towards not only the details of timber trade, but also issues faced by the intended end users—specifically the enforcement agencies.

**Fig 3:**
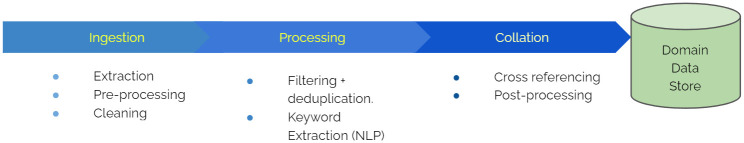
Workflow pipeline for domain knowledge module.

We created a domain-specific knowledge module that enables additional domain-specific datasets to be incorporated easily. Fig 3 indicates our workflow pipeline for the domain knowledge module, which involves ingestion of relevant datasets, keyword extraction using natural language processing (NLP), and data collation. For forest product and wood-specific domain knowledge, we curated and assembled data from the following sources: CITES Appendices [30], IUCN Red List (The International Union for Conservation of Nature Red List of Threatened Species) assessments [31], information on known and reported forest product harvest or export restrictions [32], manually curated lists of high-risk species from World Wildlife Fund (WWF) publications [8, 33], working list of commercially traded wood species [34], as well as additional relevant information extracted from these sources such as scientific names, aliases, lists of common names in multiple languages, and any range state (country) information that could be extracted from the aforementioned datasets. These data were extracted, processed, and collated to obtain a set of high risk wood species with complete taxonomic information (family, genus, species, and multiple common names) and other relevant keywords. This was a challenging task as data are sparse and distributed, and nomenclatures are often incomplete or have multiple conflicting versions. The resultant domain knowledge repository includes data on globally traded wood and forest products and is not country or region-specific, thus allowing it to have utility and relevance beyond the scope of the specific country trade data used in this proof-of-concept. In addition, we took a domain-agnostic approach to developing the workflow pipeline for this module and thus these datasets could be replaced with other data specific to a different commodity group or contain additional information specific to the supply chain trade node location where the system will be deployed. The details of this domain knowledge ingestion pipeline is presented in Algorithm 1.

**Algorithm 1** Domain Knowledge Ingestion Pipeline

**Input**: Data Sources

 Processing Constraints

**Output**: Domain Knowledge Data Module

 **Step 1**: Ingest HS Code data

 1.1 Collect HS Code definition files, from multiple sources.

 1.2 Address issues, perform initial preprocessing on each file. Address time-series shifts and changes to 6-and 10-digit HS Codes.

 **Step 2**: Process HS Code data to obtain keywords including common names, scientific names.

 2.1 Utilizing NLP techniques such as *n*–*gram* extraction obtain phrases, filter using regular expression.

 **Step 3**: Process expert curated High Risk Species data

 3.1 Clean column texts: parse region of origin, genus name, species name

 **Step 2**: Process curated data on known and reported forest product harvest or export restrictions

 3.1 Clean column texts: parse

 **Step 4**: Process IUCN Redlist

 4.1 Filter out species that are Least Concern.

 4.2 Clean text columns: Common names, IUCN Status Code, genus, species

 **Step 4**: Process CITES Data. Clean ISO codes.

 **Step 5**: Collation of data sources.

 5.1 Merge data obtained from different sources, by grouping based on scientific names.

 5.2 Perform disambiguation through automated checks, and de-duplication.

 5.3 Remove specific plant(timber) families that are not of interest, based on expert input.

 5.4 Filter to retain specific commercially traded species, based on expert input.

 **Step 6** Create a data-store with HS Codes and associated keywords comprising of scientific names, common names. Indicator flags are set HS Codes a correspond to timber species or type of interest.

 **Step 7** Extract additional information for US HTS that require Lacey Act Plant and Plant Product Declarations

**Fig 4:**
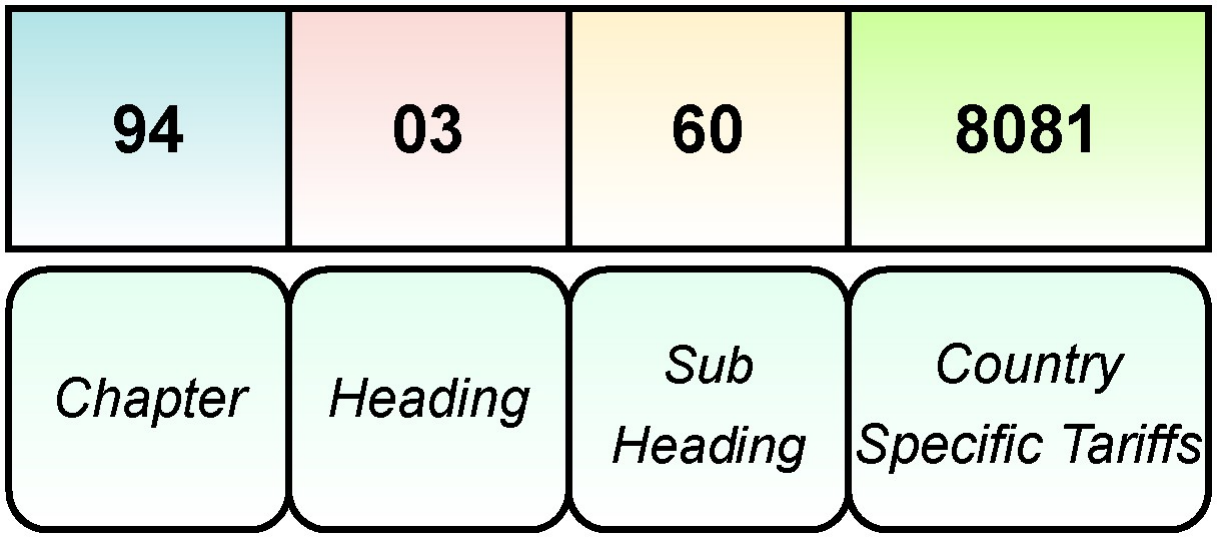
HS code nomenclature: Digits 1 and 2 (Chapter), 3 and 4 (Heading), 5 and 6 (Sub heading) are set by the WCO, whereas digits 7-10 are set by individual countries.

Official product classification descriptions of the ten-digit US HTS codes, as well as the six-digit standardized HS product nomenclature and classification system codes on which the US HTS codes are based, enumerate all products in global trade. The codes themselves can be up to 10-digits long, but only the first 6-digits are standardized and consistent across all countries. The World Customs Organization sets the standard for the 6-digit product codes and descriptions (and issues revisions on 5 year cycles —e.g., 2017, 2022) and then individual countries elect to institute more specific product descriptions utilizing 4 additional digits (digits 7-10, see Fig 4). It is a hierarchical classification, where the first two digits of the HS/HTS code present a broad product category and then each subsequent pair of digits narrows down and specifies more clearly different product categories. The HS and HTS code nomenclature system was not developed with the idea of specifying the taxonomy and scientific names (e.g., family, genus, species) of specific natural resources that are traded, but instead was developed to enable border taxes and tariffs to be levied at the time of import and export. Hence, HS and HTS product classification distinctions have to do with other product attributes and characteristics, rather than species clarity—with the lack of taxonomic specificity of HS codes for plants and forest products being particularly noteworthy [35].

Nevertheless, wood and forest-product specific HS and HTS codes and their respective descriptions at all resolutions were collected, cleaned, and processed to obtain associated keywords. We collated HS and HTS codes from multiple sources covering any nomenclature and description shifts that took place between 2015-2017, with any available taxonomic identifiers being keyword extracted [36, 37]. Despite creating a pipeline to process and incorporate the full 10-digit US HTS codes into our workflow so that US government agencies could take advantage of this pipeline, we only used the first six digits, the HS codes and their descriptions, as one of the limitations of purchased US BoL data is the lack of complete HTS codes [20].

The HS and HTS codes are used as one avenue, in addition to keyword matching, to connect the underlying trade data to the forest sector-specific domain knowledge described above. The HS and HTS codes are utilized in multiple stages. Firstly, trade records are selected to filter for all resolutions of HS and HTS codes that are known to contain wood and forest products. Using regular expressions and n-gram based keyword matches on the text descriptions associated with HS and HTS codes, we developed sets of plausible high-risk wood species imported under each code.

After both the Domain Knowledge and the Machine Learning modules are run and the records scored, specific human-defined filters based on HS and HTS codes are also applied to highlight actionable records. For example, subsets of HS and HTS codes that are specifically named as part of supply-side regulations such as log export bans [32], and demand-side regulations such as those US HTS that require Lacey Act Plant and Plant Product Declarations [10] were also collated. Thus, if a record has an HS or HTS code matching any one of the human-defined filters, the analyst can further investigate the record. This adds a layer of additional interpretability and actionability for the intended end-user.

It is important to note that while certain HS and HTS codes may contain high-risk species, these same codes may correspond to such a large number of species and be present in so many shipment records that simple rule-set based matching is neither analyzable nor actionable by analysts. At present, unless a wood product being imported into the US contains a CITES-listed species that is listed with an annotation that covers the product form being imported, or falls within the range of products, defined by the HTS code declared upon import, covered by the US Lacey Act’s Plant Declaration, then there is no requirement for an importer to declare all species contained in the product upon import, nor include any country of harvest information.

### 3.2 Machine learning module

The wood and forest products domain knowledge and datasets allowed us to extract relevant trade records, upon which we then sought to execute the second module made up of machine learning algorithms. Due to a lack of available annotated ground truth—data that represented a collection of records that were actually suspicious or confirmed to contain illegally harvested or traded wood that could be used to train a model, our machine learning models for anomaly detection needed to be unsupervised. Unsupervised in this case means that the algorithms need to find patterns in data that are deemed nominal or normal, and conversely to find unexpected patterns—to identify anomalous records [38, 39].

We evaluated the four BoL datasets independently to understand each of the variables (attributes) contained in each dataset, their definitions, but also their completeness, distribution, and correlation between each of the variables. For example, using the same dataset examples provided above, we determined that the Peru export BoL data had no missing data for the HS code and value variables. Whereas, for US import BoL data, 64% of the records had missing estimated value data, yet had no missing data for the weight variable.

**Fig 5:**
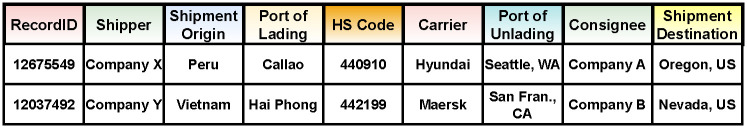
Examples demonstrating the schema of tabular bill of lading data where the attributes of interest are categorical.

**Fig 6:**
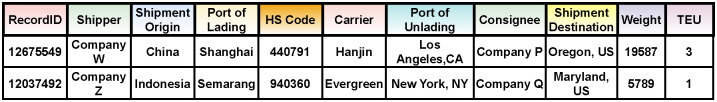
Examples demonstrating the schema of tabular bill of lading data where some numerical attributes of interest are present in addition to categorical attributes.

This meant that we needed to build two models: one model that did not rely on the value variable—and more broadly, any numerical variables—but was designed for categorical data only (Fig 5), and another model that was designed to run on both categorical and numerical data (Fig 6). Since the US government is the original collectors of US BoL data, this approach gives them the ability to choose to run their data on the model that relies on both numerical data and categorical data, though the full set of relevant and helpful data may be spread across forms collected by different agencies with varying degrees of access across each jurisdiction. Therefore, we developed two unsupervised anomaly detection models, with the characteristics of each of the underlying trade datasets dictating which model to use.

## 4 Machine learning models

### 4.1 Working with tabular data

In the data mining process, the characteristics and nature of data play a pivotal role toward the determination of applicable methodologies and the extraction of usable information. In this work, the data corpus contains tabular datasets and it is important to understand some of the challenges associated with this. Tabular data are data with rows of data instances, where each row of data comprises of instances of attribute variables in each column. Attributes are considered to be of two types—real-valued or categorical. Tabular data with only real-valued attributes is often treated as multivariate data with traditional approaches being effective. Moreover, tabular data with a mix of categorical attributes and real-valued attributes are treated as multivariate data with post-hoc encoding of categorical attributes to real-values. But in the case where the categorical attributes are of high dimensionality, such encoding is not effective. With strictly categorical attributes, there are prior works that apply itemset mining techniques [40–42]. However, these techniques are found to be not scalable for real world data, unlike many academic datasets.

**Fig 7:**
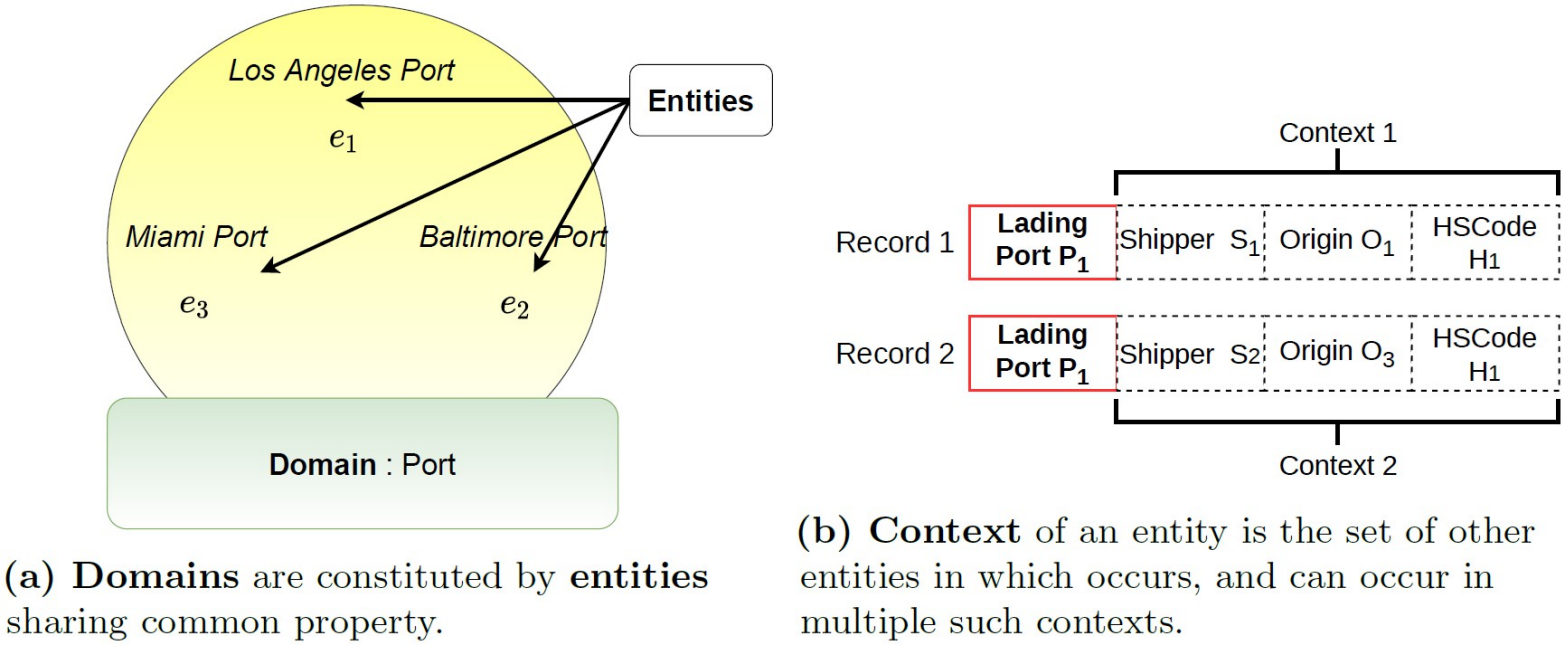
The concepts domain and entity are used to describe tabular data.

In terms of nomenclature, each categorical attribute in tabular data is termed as a *domain* and each *domain* is comprised of the set of possible values—*entities*. These are shown in Fig 7. The number of possible values a categorical variable can assume is termed as *cardinality* or *arity*. Each row is referred to as a *record*.

### 4.2 Anomaly detection

Anomalies are defined as data instances that do not conform to expected behavior or patterns in data. They are also referred to as deviants, abnormalities, or outliers in data mining literature. Anomalies have been associated with fraudulent and malicious activities in systems in multiple domains which include financial transactions, cybersecurity and healthcare. Hawkins [43] defined an anomaly as an observation that deviates so much from the other observations as to arouse suspicions that it was generated by a different mechanism.

The techniques for anomaly detection or outlier detection often overlap with the approaches employed in novelty detection. The goal of novelty detection is to find data instances that are thus far unobserved and modify the data distribution to account for these to be considered as expected. Novelties, however, are not considered as anomalies in the literature. While the terms outliers and anomalies are used interchangeably, one can delineate the distinction between the usage in certain cases. Data often contain noise and points that do not readily conform to the underlying assumptions made when creating a model are termed outliers. In certain cases, outliers indicate the inability of a model to properly capture the details of data distribution, and in other cases, they indicate the presence of noise.

The term anomaly is generally application-specific and is tied closely to the concept of outliers. Instances are of interest when they are found to not conform to the majority of data characteristics. For instance, fraudulent financial records or malicious network activities stand out from the majority of regular data instances [23, 24, 39]. In our work, therefore, we use the terms anomaly and outlier interchangeably, since our focus is to find instances that are unexpected.

The anomaly detection approaches presented in this work can be classified as model-based approaches [44], rather than proximity or density-based. Based on the availability of labels, there are three major approaches. First is supervised learning, where anomalies and nominal data have labels and can be used to train a machine learning model with a classification objective. The second approach is unsupervised anomaly detection where no labels are available. The objective is to assign scores or labels to data points such that anomalies are scored low (or high based on the scoring paradigm used) and nominal data points are scored high. Many prominent algorithms such as Local Outlier Factor [45], One Class Support Vector Machines [46] and Isolation Forests [47] fall in this category. The third class of approaches is semi-supervised, where labeled normal data is available but no labels or instances of anomalies are available. Many prior works use the terms unsupervised and semi-supervised without a clear distinction. The approaches presented in this work follow a primarily semi-supervised approach.

### 4.3 Anomaly detection approaches in tabular data

Anomaly detection in tabular data is an important task with a myriad of real-life applications, due to the presence of such data in a multitude of scenarios. Many general approaches to anomaly detection work well in most cases, where the attributes are real-valued —such as One Class SVM [46], Local Outlier Factor [45] and Isolation Forests [47]. The reason for this is that tabular data with binary or real-valued attributes can be treated as multivariate real-valued data. For a detailed survey on general anomaly detection approaches the reader can refer to survey papers [48–50].

However, tabular data with *categorical* or *discrete* attributes—especially with *high cardinality categorical attributes*—present a unique challenge. Applying encoding approaches generally used in machine learning—such as one-hot (1-0) encoding—leads to high dimensional and sparse multivariate data. Most machine learning models do not perform under these circumstances, due to what is known as the *curse of dimensionality*. Therefore, specific approaches are required for data with such characteristics.There has been prior work on handling tabular data with strictly categorical data. These include probabilistic approaches [51, 52] and information theoretic approaches such as *KRIMP* [40], *CompreX* [42] and ODD [41].

### 4.4 Multi-relational embedding based anomaly detection

In this section we describe the anomaly detection model used for tabular data with strictly categorical data—**M**ulti-relational **E**mbedding based **A**nomaly **D**etector (MEAD) [53].

The model architecture consists of a single embedding layer so that all entities belonging to all domains are represented in the same latent space. The *entity embedding* is a transformation *f*_*j*_ for the *j*^*th*^ domain i.e. attribute (column), realized using a neural network layer. Specifically, embedding of the entity in record *r* belonging to the *j*^*th*^ domain be $f_{j}\left(e_{j}^{r}\right)$, is $x_{j}^{r}$. Each domain also has an associated *weight*–*W*_*j*_—which is trained. A non-linearity on the square of the Euclidean (*L*_2_) norm of the resulting weighted entity embedding to obtain the likelihood of occurrence, or score, of a record *r*, as shown in Eq (2).

$$\begin{array}{c} z_{j}^{r}=W_{j}⊙x_{j}^{r};\;z_{r}=({\left\|\Sigma_{j=1}^{l}z_{j}^{r}\right\|}_{2}{)}^{2} \end{array}$$ (1)

$$\begin{array}{c} P_{\theta }\left(r\right)=\text{tanh}\left(z_{r}\right) \end{array}$$ (2)

The model training objective is as described in Eqs 3 and 4.

$$\begin{array}{c} L=-(\sum_{r\in R}(logP_{\theta }\left(r\right)+\sum_{k\in r^{\prime }}log(\text{tanh}\left(z_{k}^{-1}\right))))+L_{Z} \end{array}$$ (3)

$$\begin{array}{c} L_{Z}=\sum_{r\in R}\frac{1}{\left|l\right|}\sum_{j=1}^{l}\left(1-\right\|z_{j}^{r}{{\|}_{2})}^{2} \end{array}$$ (4)

**Fig 8:**
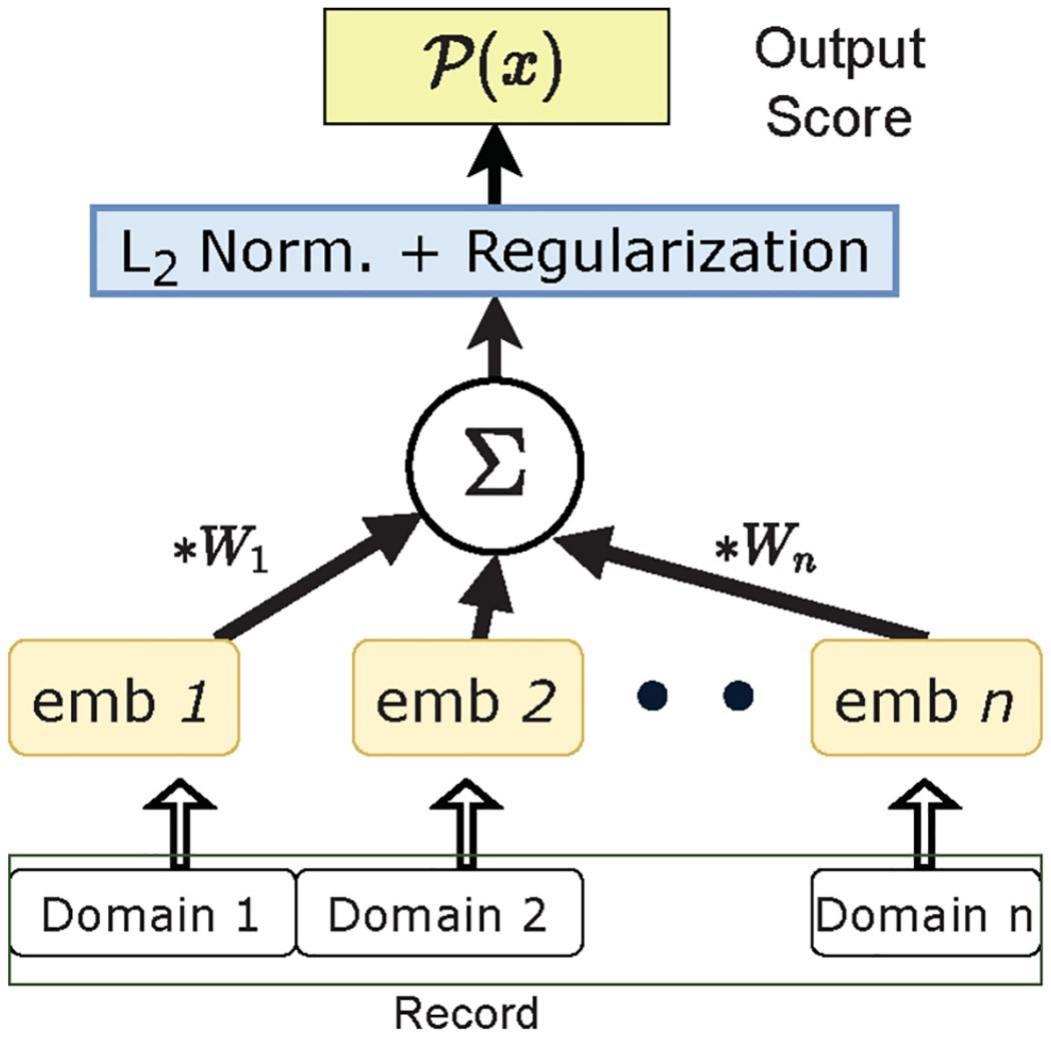
Model architecture of the multi-relational embedding based anomaly detector.

The overall architecture is shown in Fig 8. This approach ensures that embeddings of different domains which co-occur together are similarly aligned in the latent representation space. The intuition is that the weighted sum of entities corresponding to valid transactions should be additive in nature. MEAD is a likelihood based model, with records with high scores deemed normal, and records with low scores considered anomalous.

In utilizing unsupervised anomaly detection models, there are two widely accepted approaches. The first approach is to either use a *threshold* to choose the lowest scored records—in terms of likelihood, or the highest scored records in terms of anomaly scores. The second approach is to select the top *k* anomalous records. Since it is more difficult to determine an application scenario-based threshold, we utilized the second method.This approach is well suited to a larger number of datasets pertaining to different countries, where BoL data contains strictly categorical attributes.

### 4.5 Contrastive learning based anomaly detection in heterogeneous tabular data

In this section we discuss **C**ontrastive Learning based **H**eterogeneous **A**nomaly **D**etector [54]. This anomaly detection model works with tabular data, where both high dimensional categorical attributes as well as numerical attributes are present—unlike *MEAD*, which is intended for tabular data with categorical attributes only.

**Fig 9:**
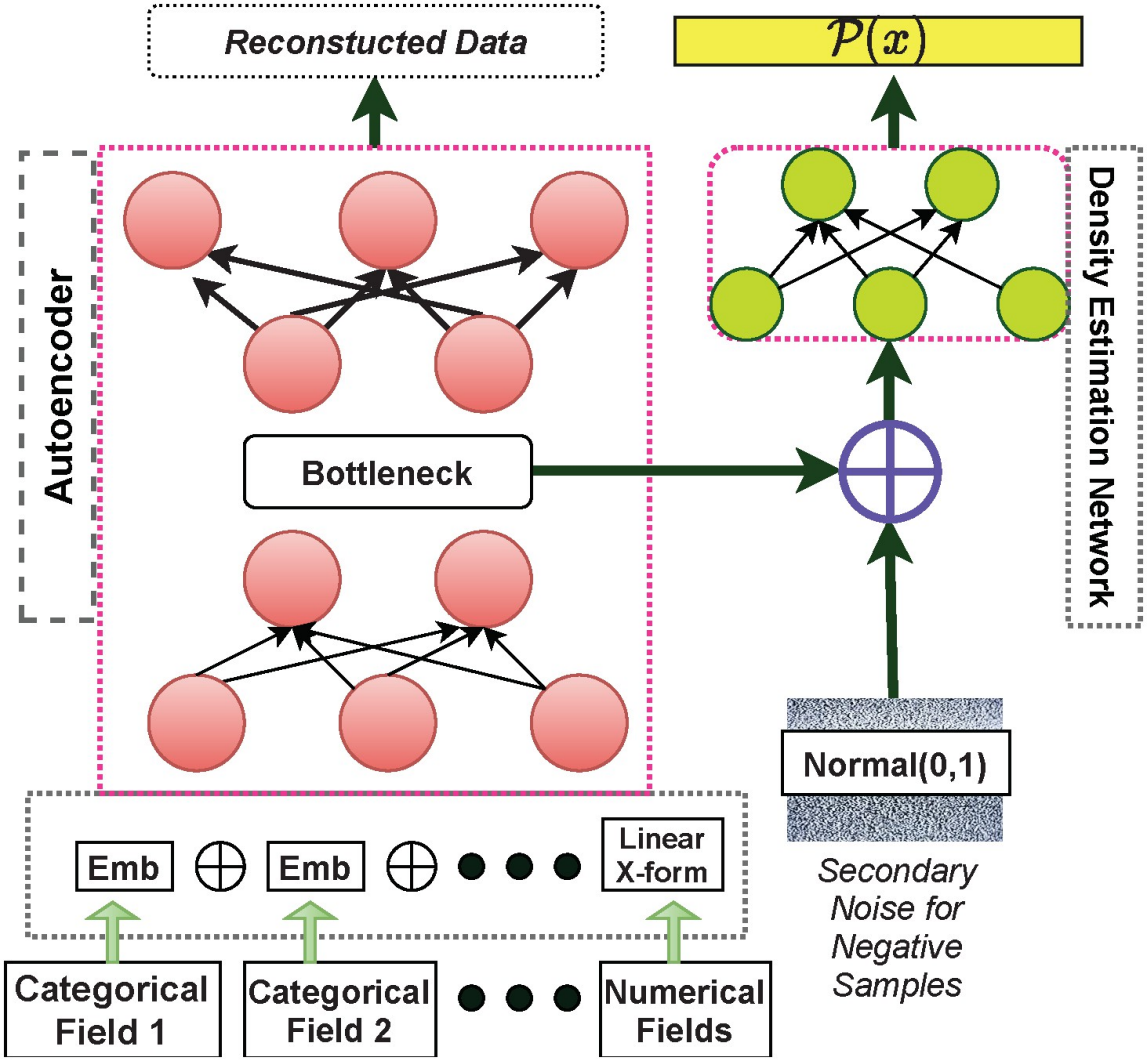
Model architecture of the contrastive learning based heterogeneous anomaly detector.

The model architecture is shown in Fig 9. The objective for training the model is based on Noise Contrastive Estimation, which allows a more direct approach towards estimating the data distribution. *CHAD* does not require hyperparameters such as number of clusters, or make assumptions about shape of clusters in latent space.

The CHAD architecture has two parts—1. Asymmetric autoencoder 2. Density estimation network. The asymmetric autoencoder has an encoder and decoder. The decoder is a fully connected dense neural network with dropout. The first fully connected layer in the encoder is a concatenation of embedding (linear transformation) for each categorical attribute (domain), with either linear or identity transformations for the set of all continuous features. The autoencoder is designed to optimize reconstruction of the input vector, which is an auxiliary task in our case since we are interested in the latent representation.

The density estimation network estimates the likelihood of records, where a higher likelihood means a normal record (i.e., not anomalous). The training objective of the density estimation network is described in Eq 5. Here *C*_*i*_ = 1 and *C*_*i*_ = 0 refer to normal records, obtained from the training set and negative samples that are generated respectively. *θ* are the trainable model parameters.

$$\begin{array}{c} \begin{array}{c} L\left(\hat{\theta }\right)=\text{arg}\;\underset{\theta }{\text{max}}\;\sum_{i}C_{i}ln(P\left(C=1|x_{i};\theta \right)\\ +\left(1-C_{i}\right)ln(P\left(C=0|x_{i};\theta \right)\\ \approx \sum_{i}ln\left(f\left(x_{i};\theta \right)\right)+ln(1-\frac{1}{\left|k\right|}\sum_{k}(f\left(z_{k};\theta \right)) \end{array} \end{array}$$ (5)

The autoencoder is trained first, then both the networks are trained jointly, followed by fine-tuning the density estimation network only. Like *MEAD*, *CHAD* is a likelihood based anomaly detection model.

## 5 Evaluation and results

Here we provide details and commentary for a small subset of ranked results from 2015-2017 BoL historical trade data. We built an internal website interface to display and comprehend the results as well as make it easier to provide a demonstration of outputs and results. Due to strained US-China relations since 2018, BoL data providers could not provide more recent China trade data, and thus we decided that of the four trade flows our models were run on, we focused on scrutinizing results from the two datasets that future BoL data could be obtained: US imports and Peru exports.

What follows are the results and analysis on the first and second highest ranked records for US imports, and the top three ranked records for Peru exports to the US. The results are ranked anomalies that have not been validated with respect to their actual level of suspicion, let alone legality. Thus, our results should not be interpreted as an indication that the given record is deemed suspicious, but rather that it was deemed anomalous and is therefore flagged for further investigation.

### 5.1 Quantitative model evaluation

The models that are utilized for detecting potentially suspicious timber transactions are *anomaly detection* models. The models MEAD [53] and CHAD [54] have been evaluated with synthetic anomalies that are injected into real test data, as is the standard approach with unsupervised anomaly detection. The general practice is to generate a set of anomalies—based on an understanding of what *anomalies* are for the specific application scenario—with the input of domain experts. We followed prior work in this regard, and generated synthetic anomalies with different characteristics using trade records from the real data set.

Since anomalies in tabular data can be described as unexpected co-occurrences and patterns, we generated synthetic anomalies based on that notion. The algorithms utilized in this generation process are explained in detail in the works that present the models [53, 54]. The models are evaluated using the precision-recall curve, specifically Area under the Precision-Recall curve(AuPR) or Average Precision—which is a widely adopted metric for evaluating anomaly detection model performance [49]. For this quantitative evaluation, we have generated labels for the synthetic anomalies and consider the test set consist of normal records. Thus, we can obtain a Precision-Recall curve. For the actual analysis, as presented in the next section—we select the top-k lowest scored records i.e. most anomalous records. Here *k* depends on the user budget; for our results, *k* was chosen to be 100, though it could have been chosen higher based on a budget analysis. The understanding is anomalies or suspicious trades are very rare, and such a reasonable value provides adequate coverage such that recall is high.

We compare the models CHAD and MEAD against competing baselines, which include current anomaly detection models that are applicable to the respective scenario. We demonstrate that our models perform comparably or favorably and thus are an optimal choice for the task. Additionally, we perform an evaluation of model run-time, and demonstrate that our models are highly efficient. Readers can refer to our more technical manuscripts describing each model with details on experimental evaluation and analysis, which we omit here for brevity [53, 54].

**Hyperparameters** Setting hyperparameters for unsupervised anomaly detection is a challenging task, since there is no validation set and labels to perform hyperparameter tuning. Both MEAD and CHAD are not sensitive to the value of hyperparameters, and during experimental evaluation, we find that they work well with reasonably set values. For MEAD the only hyperparameter set is the embedding size, which is set to 16. For CHAD, the number of neurons in the autoencoder is set based on the number of entities in a column as mentioned in the work [54] (ref. Sec.4.1). For both these approaches *negative samples* are generated to train the models, and 10 negative samples work well in both models.

### 5.2 Results: US imports

**Fig 10:**
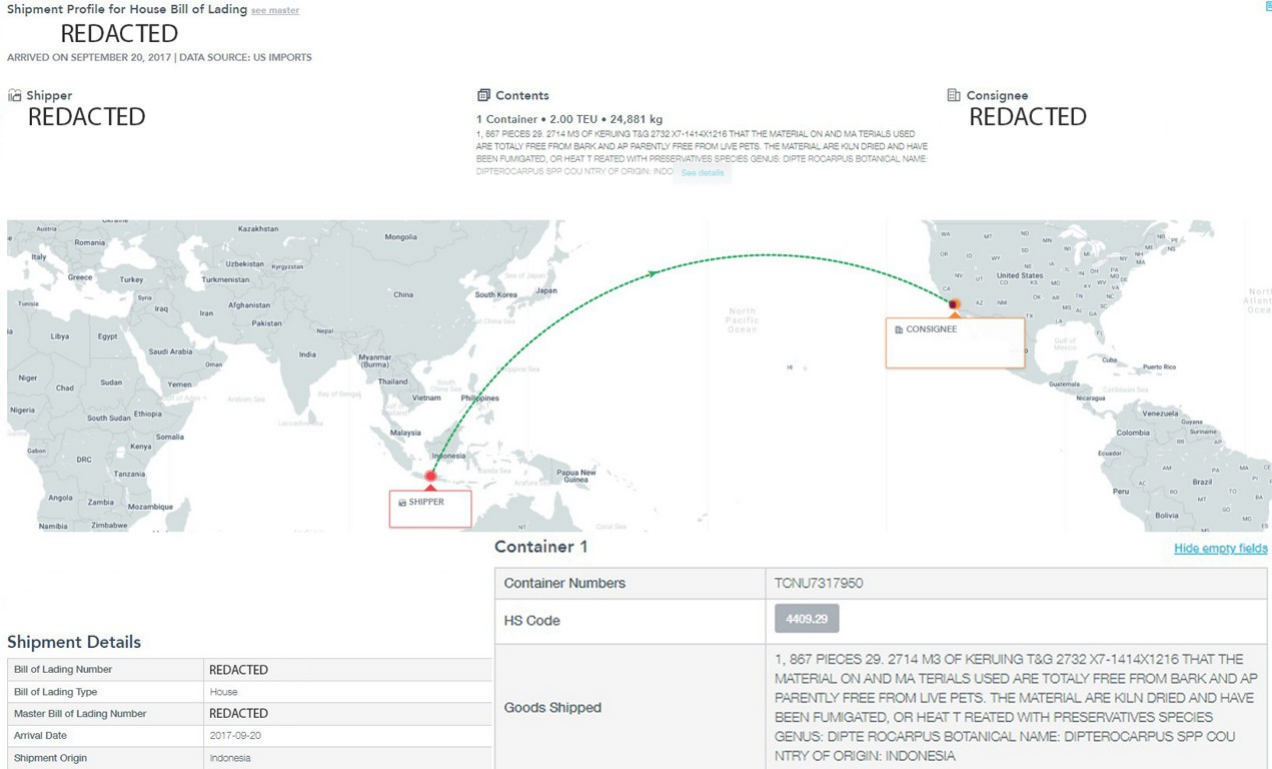
Details about the highest rank anomaly for US imports between 2015-2017 when viewed through the Panjiva interface. Reprinted from Panjiva.com under a CC BY license, with permission from S&P Global Panjiva, original copyright 2021.

From our dataset of US imports between 2015-2017, the shipment with the highest ranking arrived in the US on September 20, 2017 to the Port of Los Angeles, California. This shipment was loaded in Taiwan, but its place of receipt had been originally Indonesia—see Fig 10. The shipment comprised of one 20-foot shipping container holding 24.81 tons (approx. 50,000 lbs) of tongue and groove Keuring (*Dipterocarpus spp.*) wood under assumed Panjiva-derived HS 440929.

**Fig 11:**
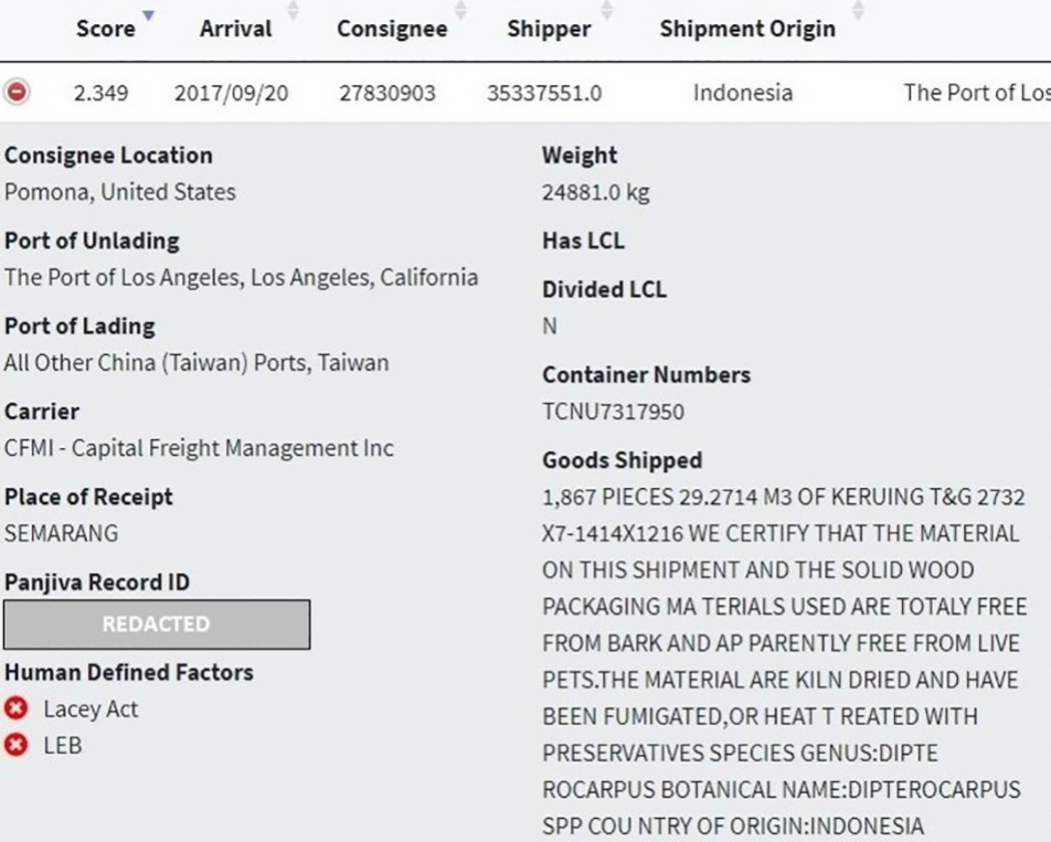
Internal website interface website showing anonymized details of the highest ranked record, with a flagging system for human-defined factors.

Keuring is a commercially valuable hardwood from Southeast Asia, with many of the individual species within the *Dipterocarpus* genus being assessed as Vulnerable, Endangered, or Critically endangered on the IUCN Red List [31]. Our human-defined factor flagging system (see Fig 11) identified that the assumed HS code (440799) requires a Lacey Act Plant Declaration Form to be filed with the US government indicating the specific species (not only genus) of the wood being imported and its country of harvest [10]. The data contained on this BoL related to genus and country of harvest (Indonesia) could be cross-referenced with the Plant Declaration Form data. In addition, our system flagged that the country of origin declared in the *Goods Shipped* field of the BoL and the shipper’s address is from a country (Indonesia, in this case) that has some type of log export ban and would therefore warrant further scrutiny by an enforcement official [32].

**Fig 12:**
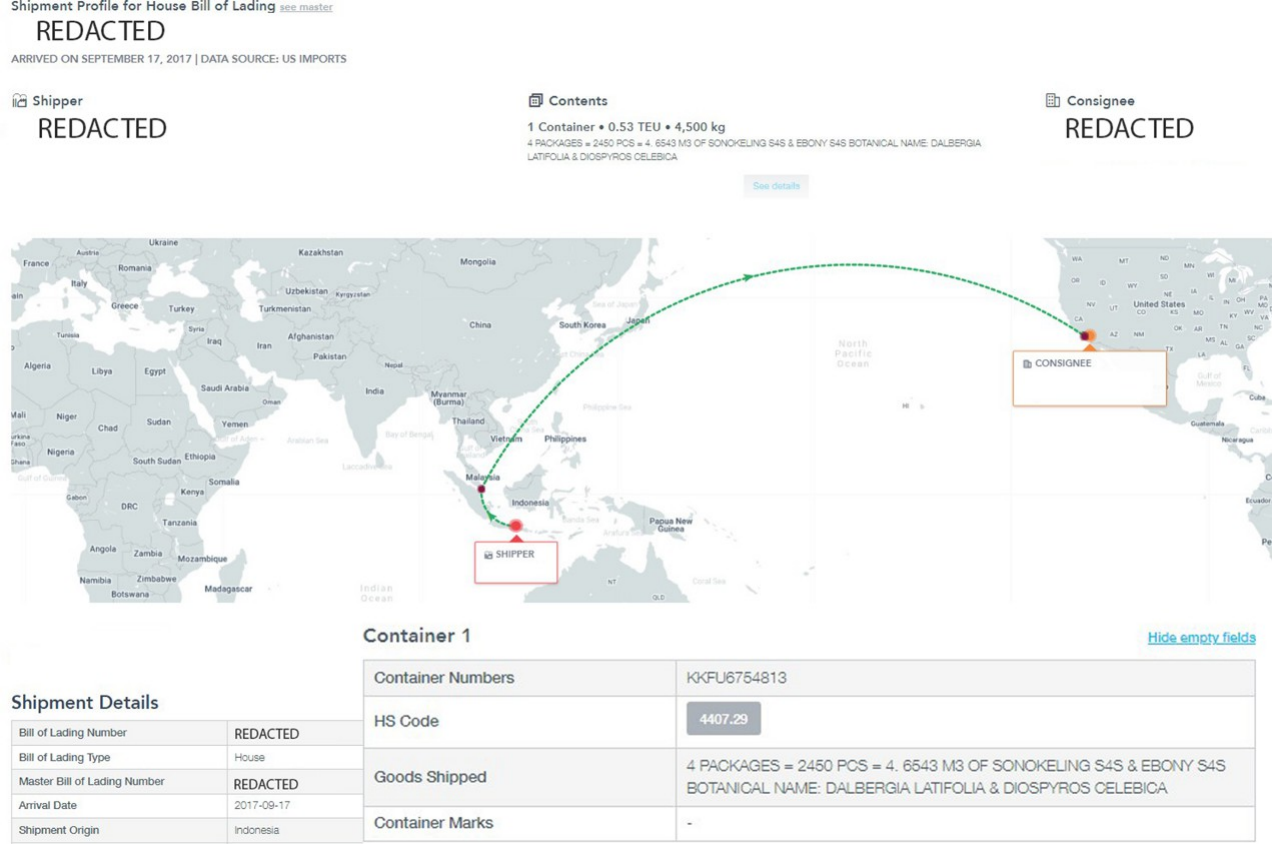
Snapshot of transaction details pertaining to the second-highest ranked anomaly for US imports between 2015-2017 when viewed through the Panjiva interface. Reprinted from Panjiva.com under a CC BY license, with permission from S&P Global Panjiva, original copyright 2021.

The second highest ranked anomaly for US imports was a shipment that arrived in the US on September 17, 2017 to the Port of Los Angeles, that had been loaded in Singapore—but its place of receipt had been originally Indonesia, as shown in Fig 12. This shipment comprised of one-quarter of a 20-foot shipping container holding 4.5 tons (approx. 10,000 lbs.) under assumed Panjiva-derived HS code 440729 of 2,450 pieces, or 4.6543 cubic meters of Indian Rosewood, also known as Sonokeling (*Dalbergia latifolia*), and Indonesian Ebony (*Diospyros celebica*).

**Fig 13:**
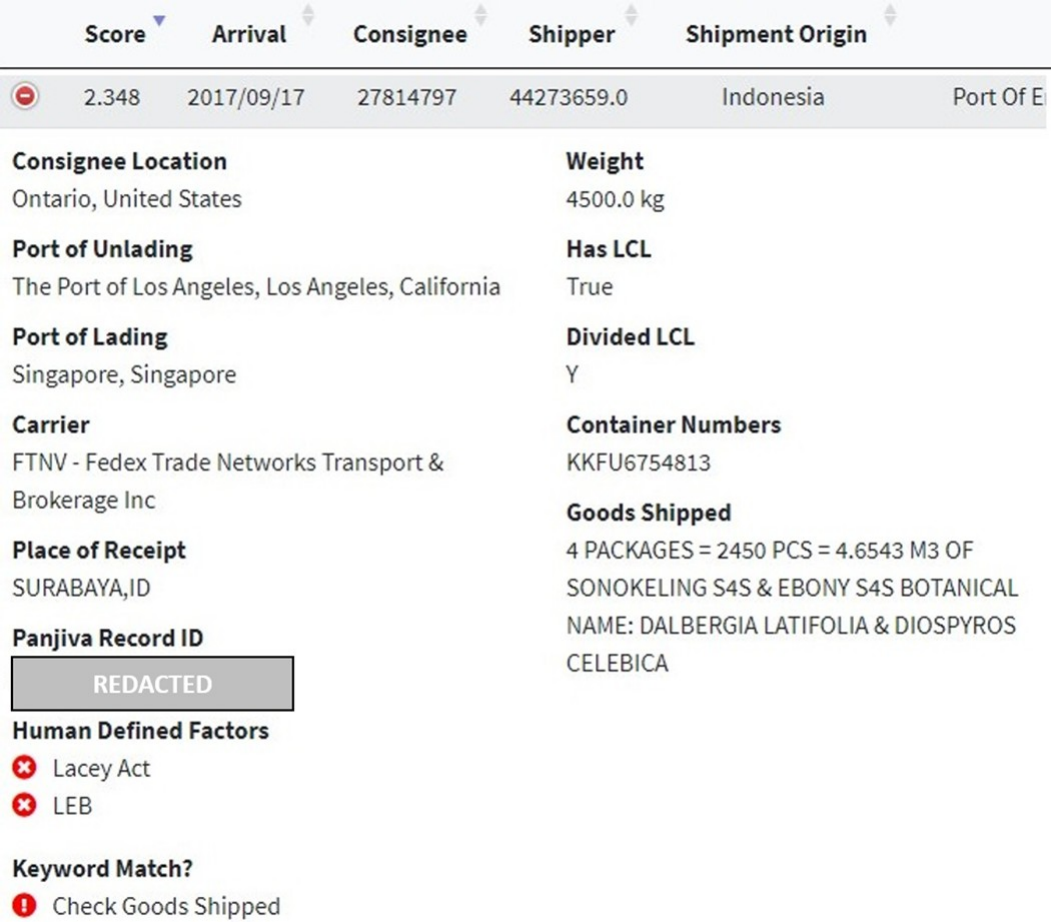
Internal website interface website showing anonymized details of the second highest-ranked record, with a flagging system for human-defined factors.

Indian Rosewood and Indonesian Ebony are commercially valuable hardwoods. As of January 2, 2017, the entire *Dalbergia* genus was listed in CITES Appendix II (in addition to Brazilian rosewood, *Dalbergia nigra*, which was already listed in CITES Appendix I). The human-defined factor flagging system identified that the HS code (440729) requires a Lacey Act Plant Declaration to be filed with the US Department of Agriculture (USDA) upon import which mandates importers to provide the species and countries of harvest for any wood contained in the product (see Fig 13). This flag allows CBP to know quickly whether importers are required to file Lacey Act Plant Declaration information with USDA, thus enabling better inter-agency communication between CBP and USDA as each agency could cross-reference any specific species and country of harvest details contained in the BoL with the declared species and country of harvest information required by USDA.

### 5.3 Results: Peru exports to the US

**Fig 14:**
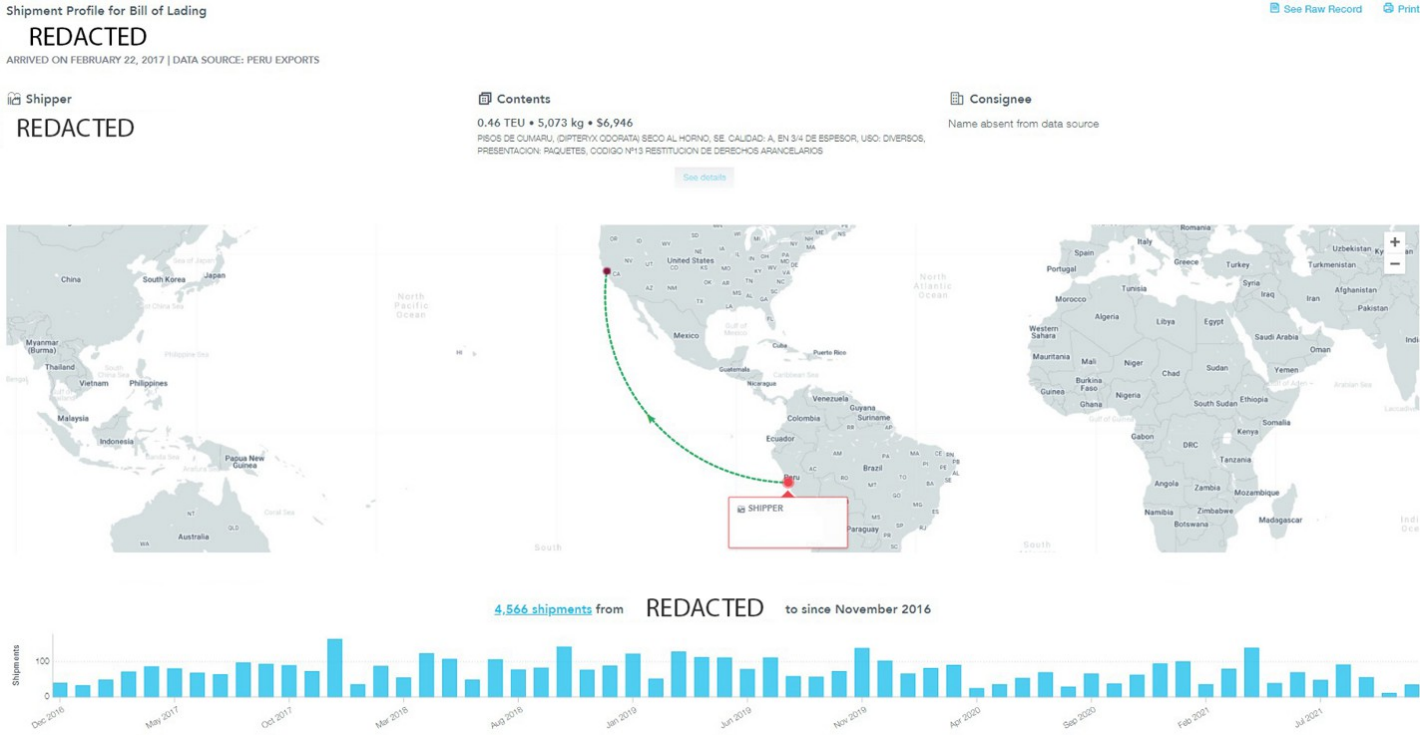
Panjiva interface indicating one of the three top-ranked records for Peru’s exports to the US, in addition to the number of shipments over time that the shipper has exported to the US. Reprinted from Panjiva.com under a CC BY license, with permission from S&P Global Panjiva, original copyright 2021.

In addition to running our models on US import data, we also ran our models on Peru export data and then filtered the results for those Peruvian shipments that were destined for the US. The top three highest-ranked anomalous Peruvian shipments all had equal scores and appeared to be connected shipments: all shared the same declaration date, were sent by the same Peruvian shipper to the same port in the US, and were given the same US-specific HS code (4409109000) by the Peruvian shipper. These three shipments had a Peruvian declaration date of February 17, 2017 in Sullana, Piura region, Peru, with a US arrival at the port of San Francisco, CA on February 22, 2017 (see Fig 14).

The three shipments contained different tropical hardwood species of interest as follows:

(i) Shipment 1 contained estoraque or Santos Mahogany (*Myroxylon frondosus*)
(ii) Shipment 2 contained Jatoba (*Hymenaea oblogifolia*)
(iii) Shipment 3 contained Cumaru (*Dipteryx odorata*)

**Fig 15:**
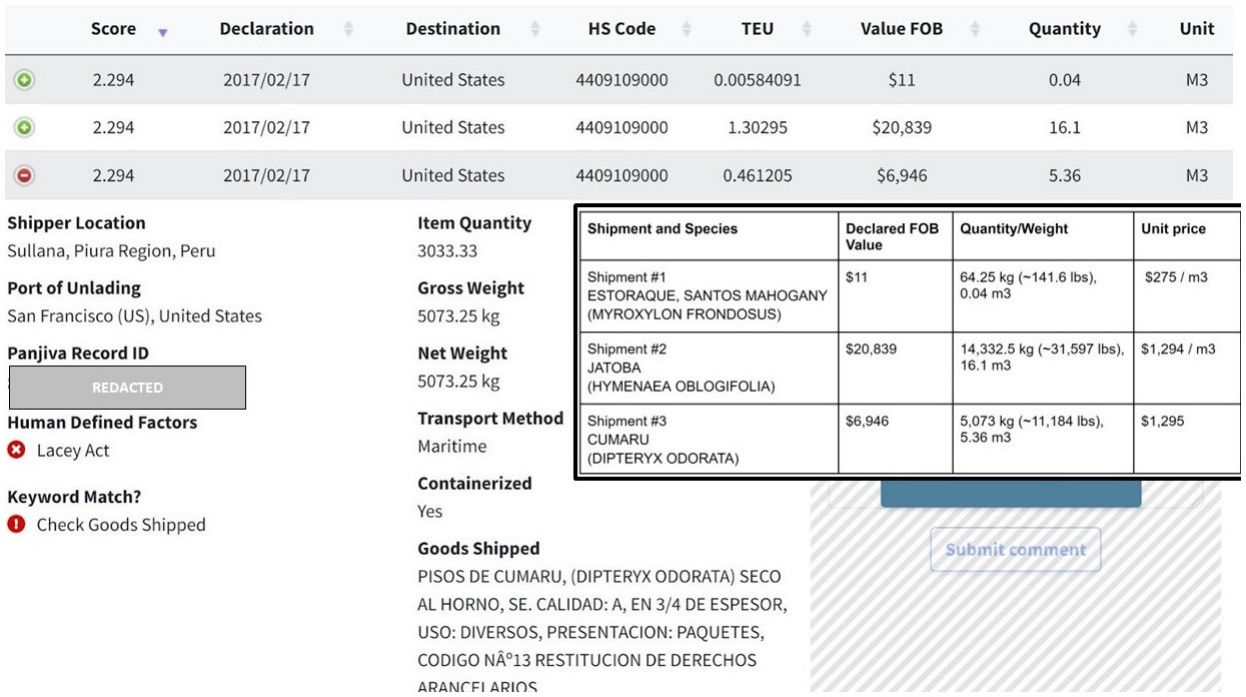
Project interface website showing anonymized details of the record, with a flagging system for human-defined factors, and subset showing the calculated unit price for each shipment.

The unit price, in US dollars per cubic meter, of each of these shipments is calculated and shown in Fig 15. Several observations can be made about this series of three anomalous records. While the shipment of Santos Mahogany (shipment 1) contained the least declared value per weight/quantity ratio of the three shipments, it does raise a red flag that a shipment of approximately 141 pounds of valuable Santos mahogany would have a declared value of $11.

Our system’s human-defined factor flagging system also identified the HS code (4409109000) for further scrutiny since any imports under this code require a Lacey Act Plant Declaration to be filed with the US government upon import. This indicates that CBP can cross-reference the specific species and country of harvest details contained in the BoL with the Lacey Act Plant Declaration data required by USDA. Furthermore, a cursory look at the first six digits of the declared HS code yields a mismatch given the tropical hardwood species named in the goods shipped fields for these three shipments. That is, the specific six-digit HS code (440910) used to export the product from Peru is incorrect, since HS 440910 refers to coniferous wood, not tropical hardwood. Such mismatches are archetypal of instances that may involve potentially suspicious trading practices and discovering them is crucial to our objective.

The results discussed above are qualitative and are obtained through analysis. Since the anomaly detection model requires data to train, and there are often pattern shifts in international trade, we design our system accordingly. In the system, we choose continuous time windows of six months of data as training sets for the model, and the subsequent month’s records are chosen as the test set. The model then scores the BoL records, with the output being a ranked list of records. We examined the top 100 records in each dataset due to a limited budget, and the aforementioned selected and presented results represent case studies demonstrating the efficacy of our approach that can be used for further investigation. It is also important to note that we were not able to obtain ground-truth labels for this proof-of-concept. The labels were not available due to real-world practicalities, including a lack of capacity for manual attention at million-plus scale data, as well as challenges with regard to obtaining security clearance which meant that annotations could not be performed by experts from government agencies who are the only personnel who have knowledge to verify illegal timber transactions. Therefore, any visual or quantitative representation such as Precision-Recall curve was not obtainable for the real-world test set.

## 6 Discussion

Our work contributes toward a greater understanding of building decision-making systems that utilize machine learning to solve challenges in real-world application scenarios. We are encouraged by the results from this proof-of-concept framework. We developed an end-to-end framework with modules to manage and query large-scale trade data, and developed human-defined factor filters and machine-learning algorithms that can be applied to different types of BoL transaction-level trade data. We addressed multiple challenges pertaining to such a data corpus with large-scale tabular records and associated complexities. Nevertheless, due to the proof-of-concept nature of this research and due to the underlying challenges that persist when trying to develop and implement an integrated framework across multiple stakeholder groups, there are limitations to our current approach that motivate future research.

Our work likely does not provide clues to what type of corrupt or illegal acts may have occurred within the forest or along the supply chain. These types of clues might become evident with more robust knowledge of the supply chain actors and their suppliers. Due to the limitations of this pilot project, we could not manually curate a database of forest-to-market supply chain actor connections, nor did we find such a body of work already established and readily available for us to use. A database that documents ways that fundamental forest violations and illegal behavior might manifest themselves in trade-based activities would assist researchers and enforcement officials in designing detection systems that take these types of activities into consideration. Nevertheless, we believe that our approach and an approach that seeks to identify clues of whether corruption and illegal acts have occurred closer to the point of harvest, are not mutually exclusive to aiding the fight against illegal logging and associated trade. One area of potential further research would be to combine such approaches into one framework.

Another area of potential future research would be to reconsider our team’s assumption that suspicious and potentially illegal shipments do not conform to expected trade patterns, which in turn, informed the formulation of the research as an anomaly detection problem. Our team discussed and researched this assumption with care; however, we are aware that it may not hold true in some circumstances. Future research could explore other formulations of our research objective beyond anomaly detection.

With respect to the choice of formulating our research question as an anomaly detection problem, an area of further inquiry could be designing more robust anomaly detection systems, especially dealing with very rare entities. Other possible research directions include obtaining better performance metrics—which is a perpetual research motivation. Refining the overall system through such an in-situ evaluation process to fine tune the interaction between users, interface, and algorithm is also part of possible future work. There is room for research into improving our method and framework such as taking into account concept drift in trade records, dealing with suspicious trade instances which are non-anomalous and customizing the user interface further as per requirements of analysts. Thus our current work motivates a continuing research direction regarding anomaly detection in tabular data.

Another broad theme of future research stems from the fact that we were not able to have any human intervention for validating our outputs and results. At present, our current system only flags anomalous, not suspicious, records. To improve accuracy and refine the algorithm to target not just anomalous, but genuinely suspicious shipments, the following types of training data are needed: (*i*) ground-truthed suspicious or high risk shipments; and (*ii*) ground-truthed non-suspicious and low risk shipments. Additionally we would need to validate whether the flagged shipments (output results) are genuinely suspicious.

The software framework of our current system is built with flexible modules that follow object-oriented principles that can be extended to run on dynamic real-time data. Our current proof-of-concept modules are built using static historical datasets covering 2015-2017. Our domain knowledge module could be improved upon to use dynamic data that does not just represent a single backward looking snapshot in time, and could be expanded to incorporate a variety of other traditional and non-traditional sources to aid targeting. Several real-time auxiliary datasets would need to be updated, including yearly HS and HTS code changes, in order to run our system on real-time BoL data.

In addition, two areas of current ongoing research into unsupervised anomaly detection models that would greatly assist the usability of our models and framework are the ability to interpret why a given record is deemed anomalous, and the ability for human-in-the-loop feedback to be incorporated into the algorithms [55]. Developing a system that provides for interpretability and explainability of why a record is deemed anomalous is a non-trivial task, given the complexity of the anomaly detection models. Model explainability [56] and trust are active areas of research in artificial intelligence and machine learning [55, 57–59]. In addition, the nature of trade data and detection of potentially suspicious trade records necessitated building an unsupervised anomaly detection model that does not require human input to determine anomalous records. Nevertheless, there are circumstances when human-in-the-loop input would be advantageous -for example, including information on tip offs that an exporting company is engaged in illicit trade, or other information that is gathered through investigations could be helpful, if not essential, to flagging real-time shipments or forecasting risk of future shipments.

There are several on-going related research projects by other groups that complement our research that, if brought together, could offer a more robust framework for detection of suspicious shipments of wood and forest products—including, but not limited to, Arbor Harbor [60], TRAFFIC’s research on politically exposed persons and corruption in the wood and forest products sector using machine learning and artificial intelligence [61], and the ILAT Risk tool being developed by Forest Trends and Environmental Investigation Agency [62].

## 7 Conclusion

This proof-of-concept research has many potential application scenarios with similar data characteristics. While we incorporate elements of domain knowledge and requirements, these are generalizable and can be adapted to other scenarios since the underlying machine learning models are not dependent on the application scenario. Therefore, this work has the ability to catalyze future research to advance the use of data science in supply chain and trade data research, with lessons that can be applied not just to further illegal logging and associated trade detection, but also to research that seeks to detect illegal wildlife, and illegal, unreported, and unregulated (IUU) fishing, and other commodity trade where identifying anomalies and suspicious shipments might assist government agencies—and ultimately, help protect ecosystems from illegal resource harvesting and associated trade. We envision broadening our initial paradigm of TimberSleuth [55] to a more generalizable TradeSleuth research program.

The work presented here also addresses important research questions beyond the scope of the immediate application scenario of detecting anomalous forest product shipments. While tabular data is ubiquitous and one of the most general forms of data, mining such data has multiple associated challenges and it has not been the focus of the majority of research on natural language, image, and graphs. Our work addresses an important and challenging problem scenario of dealing with a tabular data corpus with high dimensional categorical variables, along with mixed variables in certain cases. Due to the lack of a well defined relational or semantic structure in such data—finding patterns is non-trivial, especially in the absence of ground truth data which is true for most real world application scenarios. Moreover data instances in tabular data or records are not atomic, but rather are comprised of a set of variables with their own inherent distributional characteristics. Data mining on such tabular data therefore entails comprehensively capturing the patterns on these complex data instances.
